# Changes in sports gambling behavior during the COVID-19 pandemic in Canada

**DOI:** 10.3389/fpsyt.2022.1018234

**Published:** 2022-11-09

**Authors:** Elijah Otis, Andy J. Kim, Sherry H. Stewart, Simon B. Sherry, Igor Yakovenko

**Affiliations:** ^1^Department of Psychology and Neuroscience, Dalhousie University, Halifax, NS, Canada; ^2^Department of Psychiatry, Dalhousie University, Halifax, NS, Canada

**Keywords:** gambling, COVID-19, availability hypothesis, Canada, adults, sports betting

## Abstract

Sports betting is one of the most popular forms of gambling in Canada; recent prevalence estimates indicate that 7.9% of Canadian adults endorsed gambling on sports in the past year. The ongoing COVID-19 pandemic led to the temporary closure of most major sports leagues worldwide beginning in March of 2020. These sudden closures created a dramatic decrease in the availability of sports betting opportunities in the early stages of the pandemic, followed by a subsequent increase in availability as most sport leagues returned during the summer of 2020. Using a retrospective self-report measure of gambling participation, the present study investigated how the gambling behaviors of *N* = 85 past-year sports gamblers changed over the course of the pandemic. It was hypothesized that sports gamblers would report an initial decrease in gambling behaviors from pre-pandemic baseline levels to the early stages of the pandemic in May of 2020 when the availability of sports gambling was heavily restricted, followed by an increase in gambling behaviors from May to August, in accordance with the re-emergence of live sporting events. The general pattern of results supported the hypotheses, though gambling behaviors did not completely return to baseline levels. Beyond quantifying the changes in gambling behaviors over the early stages of the pandemic in Canada, results may have implications regarding the utility of voluntary gambling exclusion programs as well as legislation concerning gambling access.

## Introduction

Sports betting, broadly defined as the wagering of money on the prediction of some outcome of a sporting event (1), is one of the most popular formats of gambling, both worldwide and in Canada (2). Sport gamblers tend to be overwhelmingly young men in the 18–34 years age range (3, 4). Sports bettors are at particularly high risk for problem gambling, particularly when motivated by monetary, socialization, and enhancement motives (5, 6). Some structural elements of sports betting can carry especially high risk for problem gambling, including engaging in live in-play wagers (i.e., betting on the outcome of an event in progress), betting on micro-outcomes of sporting events, and betting on Daily Fantasy Sports contests (7–10).

### Availability hypothesis

The “availability hypothesis” posits gambling involvement is closely tied to the availability of gambling opportunities in an environment, such that increases in the availability of gambling opportunities lead to increases in gambling participation and, by extension, increases in the prevalence of problem gambling (11–14).

One limitation to most of the research on the availability hypothesis is that almost exclusively, studies focus on the impact of *expansion* of gambling availability on gambling involvement. This leaves open the question of what happens to gambling involvement in response to the *reduction* of available gambling opportunities. This is likely in part due to the fact that the past several decades has seen almost universal expansion of legal gambling availability, leaving few opportunities to study how gamblers react to decreases in availability (15). One such rare opportunity occurred in 2007, when Norway instituted a complete ban on virtually all electronic gaming machines (EGMs), a form of gambling which previously dominated the Norwegian gambling market (16). Lund (17) studied the impact of this ban on Norwegians’ gambling behaviors and found a sharp decline in the prevalence and frequency of EGM play after the ban, as well as in the prevalence of problem gambling.

### Disruption of sports betting due to the COVID-19 pandemic

The ongoing COVID-19 pandemic had a profound impact on the availability of sports betting. The World Health Organization (WHO) initially classified COVID-19 as a pandemic on March 11th, 2020 (18). In the wake of the WHO declaration and global implementation of pandemic control measures, virtually all major sports leagues worldwide suspended play or postponed events indefinitely from March to April 2020 (19).

By May 2020, a limited number of major sports leagues had begun to resume holding live sporting events, including the UFC (May 9th), NASCAR (May 17th), and the Bundesliga, the top tier league in German soccer (May 16th). By the beginning of August, most major sports leagues with large global followings had resumed play, including Major League Baseball (July 23rd), the National Basketball Association (July 30th), and the National Hockey League (August 1st), allowing for sports bettors to resume wagering on some of the sports leagues with the largest international audiences.

Given the widespread popularity of sports betting, the sudden scarcity of live sporting events on which to gamble in the early months of the COVID-19 pandemic presented a rare opportunity to observe how sports gamblers’ gambling behaviors were affected, akin to the natural experiment investigated by Lund (17) in the wake of EGM bans in Norway. The resumption of sports leagues after the restrictions also provides an opportunity to examine whether the returned availability of betting opportunities led sports betters to resume their pre-pandemic levels of betting behavior, consistent with the availability hypothesis.

Since the onset of the pandemic in March 2020, several studies have investigated the impact of the COVID-19 pandemic on gambling behaviors during the early months of the pandemic, with multiple reviews finding an overall reduction in gambling behaviors during the first lockdown period, consistent with the availability hypothesis (20–22). Given the incongruent manner by which early pandemic public health restrictions reduced access to some forms of gambling (e.g., live sports betting, land-based casinos) compared to other forms of gambling (e.g., online casino games), it is worthwhile to consider whether gamblers with reduced access to their primary modality of gambling during lockdown (e.g., sports gamblers) responded by substituting their gambling toward other modalities that were more accessible. Additionally, there has been some speculation that the temporary absence of traditional live sporting events may have fueled an increase in the popularity of electronic sporting events (e.g., online video game competitions), for traditional sports fans and sports gamblers alike (23). Evidence regarding the existence of such a “substitution effect” has been mixed in the extant literature, with some studies finding an increase in the prevalence and expenditure in online casino gambling during the initial lockdown period, suggestive of a substitution effect (24–26), and others finding no such evidence (27–29).

Despite sports betting being the modality of gambling perhaps most restricted by early lockdown measures, only three known studies have specifically investigated changes in sports gamblers’ gambling habits during the COVID-19 pandemic. Auer et al. (27) found an overall reduction in online expenditure among sports bettors from February 2020 to April 2020, and no evidence for substitution from sports betting to other forms of gambling, such as online casino games. Conversely, Wardle et al. (30) found that a significant minority of sports bettors in their sample initiated new forms of gambling during the lockdown. Moreover, nearly a third of sports bettors increased their frequency of gambling on at least one gambling activity during lockdown, suggesting the possibility of some substitution toward gambling modalities which remained easily accessible. Similarly, Nosal and Lopes-Gonzalez (28) found that 87.5% of Polish sports bettors surveyed in early May 2020 had substantially reduced their gambling compared to before the pandemic, though their results also indicated some evidence of substitution toward gambling on e-sports and non-traditional sports leagues that were still operational during restrictions. Though these studies provide important information regarding the impact of the pandemic on sports bettors in a European context, it is not clear that these results necessarily generalize to North American sports gamblers. Moreover, these studies did not control for several important correlates of gambling involvement that may have influenced the outcomes such as personality traits and gambling motives (31, 32).

While a general pattern of a decrease in gambling behaviors during the early months of the pandemic has now been well established in the literature, relatively few studies have examined changes in gambling behaviors past this initial lockdown period, as restrictions limiting the availability of gambling opportunities began to ease. To date, only three known studies (26, 29, 33) have evaluated changes in gambling behaviors across multiple timepoints of the pandemic. Biddle compared gambling prevalence data from three separate population surveys on Australian adults collected during April 2019, May 2020, and November 2020. Biddle found a decrease in past year gambling prevalence, both for overall gambling and for sports betting specifically, between April 2019 and May 2020, as well as a subsequent partial rebound in gambling prevalence from May 2020 to November 2020. Similarly, a study by Fluharty et al. (26) in the UK found the majority of gamblers surveyed reported no change or a decrease in their gambling behaviors during the first lockdown period. These authors also noted a small proportion of their sample typified by greater stress, alcohol use, depression, and anxiety reported increasing gambling during this period despite the restricted availability of gambling opportunities. This troubling pattern is consistent with findings conducted in Swedish and German samples (25, 34, 35). Moreover, nearly half of the respondents in the Fluharty et al. (26) study who had reported increased gambling during lockdown maintained or increased their gambling as lockdown restrictions were lifted in early August 2020. Lastly, Månsson et al. (29) also found that most of their sample of Swedish gamblers surveyed across the first and second waves of the pandemic reported no change or a decrease in their gambling behaviors, though again, a significant minority of gamblers increased their gambling behaviors over this time frame. To date, no studies have investigated changes in gambling behaviors across multiple stages of the pandemic in a Canadian sample.

### The present study

The present study sought to address these gaps in the literature by investigating how sports bettors’ gambling habits were broadly affected by the initial suspension and subsequent resumption of major sports leagues during the ongoing COVID-19 pandemic. In accordance with previous literature (33) and the availability hypothesis (11), it was hypothesized that there would be a significant decrease from the beginning of sports league closures in February/March, 2020 to May, 2020 in both gambling frequency (number of days gambled) and intensity (total duration of gambling, total gambling expenditure), followed by a subsequent increase (rebound) in gambling frequency and intensity between May and August, 2020 when the leagues fully reopened. The present study also sought to explore the possibility of substitution from sports-based gambling modalities to non-sports related gambling across these three timepoints. Given the inconsistencies in the literature to date regarding the existence of substitution from sports gambling to non-sports gambling during the pandemic, no specific hypotheses were made for this objective. In summary, we aimed to address the following two research questions: (i) how did sports gamblers’ betting habits change in response to changes in the availability of gambling opportunities during the initial phase of the pandemic?; and (ii) did sports gamblers react to the decreased availability of sports gambling by gambling more on non-sports modalities?

## Materials and methods

### Participants and procedure

The sample consisted of sports gamblers recruited from two sources: a university student participant pool (*n* = 46) and online advertisements in the community (*n* = 54). To be eligible, individuals needed to confirm that they were: (1) over the age of 16, and (2) had gambled on a sporting event, and/or played Daily Fantasy Sports, at least once in the past 12 months.

Undergraduate psychology students were recruited through a large Eastern Canadian university’s online research participation system. Participants were given access to the survey link administered via the survey hosting platform REDCap immediately after signing up for the study online. Community members were recruited through online advertisements posted to Facebook, Twitter, and Kijiji; they were asked to contact the lab to confirm their participation and subsequently were emailed a survey link.

One hundred and ninety-one individuals responded to the survey. All participants were required to complete a brief eligibility screener before proceeding to the main survey; *n* = 34 respondents were excluded at this stage due to ineligibility. A series of data quality checks were also conducted prior to analysis, and an additional *n* = 39 respondents were excluded from analyses according to the following criteria: inconsistent responding (*n* = 11), having no data past the eligibility screen (*n* = 16), and responding more than once (identified using their IP address; *n* = 12). All participants were given the option to exclude their data from analyses upon completing the survey, which *n* = 18 participants did by responding no to the question: “do you think we should include your responses in our data?” This latter question was included in order to help eliminate those participants who may not have been paying full attention while completing the survey. These exclusions left a final sample size for analyses of *N* = 100. An institutional research ethics board approved the study. Participants provided informed consent and were compensated by a partial course credit (for university-recruited participants) or a $10 CAD gift card to Amazon.ca (for community-recruited participants). Data were collected between September and December of 2020.

### Materials

#### Demographics questionnaire

Demographic data were collected with respect to participants’ age, sex, relationship status, and monthly spending (i.e., monthly disposable income in thousand $CAD) through author-compiled survey questions.

#### Gambling timeline followback

The Timeline Followback (TLFB) method was originally developed by Sobell and Sobell (37) to increase the accuracy of retrospective reporting of alcohol consumption behaviors. The G-TLFB (36) is an adaptation of the TLFB to allow for the accurate measurement of gambling behaviors. In the G-TLFB, participants are given a calendar corresponding to a specific period and asked to first indicate on which days they gambled, the duration of their gambling for each day, and the amount of money they spent gambling. The G-TLFB was modified for the present study to also ask what type(s) of gambling (e.g., poker, sports betting) participants engaged in, for each day they gambled. Participants completed the G-TLFB for three distinct 14-day time periods. The first period, from February 26th to March 10th, 2020, was chosen to establish a pre-pandemic “baseline” of gambling behaviors in the 2-weeks prior to the WHO declaring COVID-19 a pandemic on March 11th, 2020. The second period, from May 1st to May 14th, 2020, was chosen to represent an early pandemic timepoint during which most sports leagues worldwide had suspended play temporarily (19). The third and final period, from August 1st to August 14th, 2020, was chosen to represent a later timepoint during the pandemic by which most major sports leagues had resumed play (38). To facilitate recall, participants were encouraged to use memory aids such as a personal calendar or bank statements, and referencing important events (e.g., birthdays, holidays). Participants were also encouraged to take their best guess if they could not remember the exact day on which they gambled (36).

Test–retest reliability calculated by Weinstock et al. (36) for recall of gambling variables during the past 6-months is in the adequate to high range, ranging from *r* = 0.74 to *r* = 0.96. Weinstock et al. also found that scores on the G-TLFB variables to be moderately to highly correlated with scores on a daily diary form asking participants to report their daily gambling over the same period as the G-TLFB.

#### Mini-international personality item pool

The Mini-IPIP (39) is a 20 item five-factor model (FMM) measure of personality that assesses neuroticism, extraversion, intellect/imagination (synonymous with openness to experience), agreeableness, and conscientiousness. Internal reliabilities for the subscales are adequate for short scales, ranging from α = 0.67–0.77 (39). The MINI-IPIP was included in the present study to allow for control of individual differences in the five-factor personality traits that have been consistently linked to gambling outcomes (40).

#### Problem gambling severity index

The PGSI (41) is a nine-item screener used to measure severity of gambling-related problems. The PGSI has high internal reliability (α = 0.84), as well as high concurrent validity with scores on other disordered gambling measures such as the South Oaks Gambling Screen (*r* = 0.83) (41). The PGSI was included in the survey to allow for a description of the sample in terms of overall level of problem gambling severity and to control for individual differences in problem gambling severity.

#### Gambling motives questionnaire

The GMQ (31) is a 15-item self-report measure designed to measure motivations for gambling, and is divided into three subscales measuring social motives, coping motives, and enhancement motives. Stewart and Zack (31) demonstrated high internal consistency (α > 0.80) for each of the three GMQ subscales. Moreover, the GMQ has been validated in both community-recruited adult gamblers and university student gamblers including evidence of structural, concurrent, and predictive validity (31, 42).

### Data analysis

Linear mixed models were used to evaluate all hypotheses concerning changes in gambling behaviors across timepoints on gambling behavior outcomes of interest. These outcomes were defined as follows: G-TLFB total time spent gambling measured in minutes (Duration), G-TLFB total frequency of gambling measured in days (Frequency), and G-TLFB total spending measured in $CAD (Expenditure). The present study also examined each of these outcomes in relation to sports gambling (e.g., sports betting, E-sports betting, horse race betting, Daily Fantasy Sports) or non-sport related gambling (e.g., casino games, poker). Time 1 was used to refer to the 14-day period of February 26th–March 10th, 2020 (pre-pandemic baseline); Time 2 for the 14-day period of May 1st–May 14th, 2020; and Time 3 for the 14-day period of August 1st–August 14th, 2020. To examine the potential substitution of sports gambling to other forms of gambling (non-sports), and vice versa, the opposite gambling outcome and its interaction with timepoint were entered as predictors in the model. For example, when examining Duration for sports gambling as the outcome, Duration for non-sports gambling and its interaction with timepoint (coded as Time 1, Time 2, and Time 3) were entered as predictors; significant interactions indicated substitution effects. Covariates for all models included demographic variables (age, sex, monthly spending, marital status, recruitment source) and psychological variables (PGSI score, five mini-IPIP subscale scores, and three GMQ subscale scores). These covariates were selected to control for known associations with gambling behavior. Linear mixed models were estimated using the nlme package (version 3.1-153) in R (version 4.2.1), and estimated marginal means were calculated using the emmeans package (version 1.7.2).

Models were specified using random intercepts. For each model, timepoints were entered as both a fixed factor and a random slope and analyzed as a categorical variable to allow for the examination of changes in gambling behavior across individual timepoints. Restricted maximum likelihood estimation (REML) was used to estimate parameters. The correlation structure of each model was Continuous Autoregressive (CAR1). Extreme values were winsorized rather than excluded from analyses (43).

## Results

Descriptive information for community (*n* = 54) and university-recruited participants (*n* = 46) appear in Table 1. A series of *t*-tests were conducted to determine if community participants significantly differed from university participants on continuous study variables. Chi-square tests were used for categorical variables (gender, marital status). Community participants reported significantly higher levels on age, proportion of males, monthly spending, all three gambling motives, and several gambling outcomes (for sports gambling, especially) than university participants (see Table 1). The average PGSI scores for community participants (mean = 7.30, SD = 5.38) were near the threshold for problem gambling [≥8; (44)] and at significantly higher levels than university participants (mean = 4.36, SD = 6.58). Given the differences in the samples, recruitment source was used as a control variable in linear mixed models.

**TABLE 1:** Demographics of community and university-recruited participants.

	Community participants		University participants			Total sample
	***n* = 54**		***n* = 46**			***N* = 100**
	**Mean (SD)**	***n***	**Mean (SD)**	***n***	***P-value***	**Mean (SD)**
**Demographics**						
Age	29.31 (5.91)	52	21.09 (3.82)	45	**<0.001**	25.49 (6.50)
Gender	39 (72%) males	54	24 (52%) males	46	**0.038**	63 (63%) males
Marital status	26 (48%) single	54	29 (63%) single	46	0.136	55 (55%) single
**Gambling**						
PGSI	7.30 (5.38)	46	4.36 (6.58)	42	**0.025**	5.90 (6.13)
Monthly spending	1.37 (0.96)	54	1.00 (2.97)	46	0.420	1.20 (2.13)
**MINI**						
Openness	3.44 (0.50)	46	3.54 (0.42)	42	0.333	3.49 (0.46)
Conscientiousness	3.49 (0.72)	46	3.50 (0.72)	42	0.925	3.49 (0.71)
Extraversion	3.20 (0.79)	46	3.06 (0.84)	42	0.418	3.13 (0.81)
Agreeableness	3.65 (0.64)	46	3.84 (0.64)	42	0.169	3.74 (0.65)
Neuroticism	2.90 (0.58)	46	2.76 (0.64)	42	0.288	2.84 (0.61)
**GMQ**						
Enhancement	2.98 (0.90)	45	2.18 (0.81)	42	**<0.001**	2.59 (0.94)
Social	2.68 (0.85)	45	1.92 (0.72)	42	**<0.001**	2.31 (0.88)
Coping	2.51 (1.05)	45	1.56 (0.87)	42	**<0.001**	2.05 (1.07)
**Overall gambling**						
Duration–T1	336.70 (355.23)	54	171.49 (372.89)	46	**0.029**	260.71 (375.55)
Frequency–T1	6.40 (5.18)	54	1.87 (2.94)	46	**<0.001**	4.31 (4.84)
Expenditure–T1	258.24 (373.95)	54	226.69 (726.93)	46	0.791	243.73 (561.52)
**Sports gambling**						
Duration–T1	237.15 (311.96)	54	98.62 (322.18)	46	**0.032**	173.42 (322.64)
Frequency–T1	4.86 (4.74)	54	1.20 (2.30)	46	**<0.001**	3.17 (4.22)
Expenditure–T1	153.89 (285.01)	54	164.26 (644.06)	46	0.920	158.66 (481.73)
**Non-sports gambling**						
Duration–T1	97.10 (209.38)	54	63.86 (172.13)	46	0.386	81.81 (192.91)
Frequency–T1	1.57 (2.12)	54	0.67 (1.41)	46	**0.013**	1.16 (1.87)
Expenditure–T1	100.83 (230.01)	54	36.52 (139.28)	46	0.089	71.24 (195.39)

Significant p-values (<0.05) are in bold.

Descriptive information for the total sample (*N* = 100) is in Table 1. The sex distribution was *n* = 63 male, *n* = 37 female, and ages ranged from 18 to 45 years, with a mean (SD) age of 25.49 (6.50) years. The mean monthly disposable income in thousand Canadian dollars for the total sample was 1.20 (SD = 2.13). In terms of their current relationship status, *n* = 55 participants reported being single, and *n* = 45 reported being in a romantic relationship. Fifteen participants were missing data on one or more covariates and thus were excluded from all linear mixed models (resulting in *N* = 85).

### Main effects of time period on overall gambling

**FIGURE 1:**
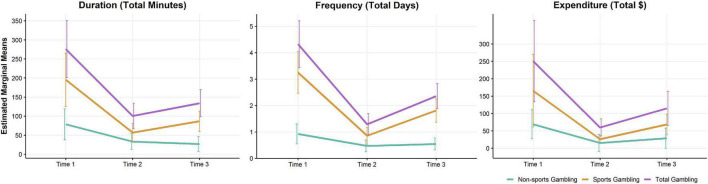
Estimated marginal means by gambling outcome. Time 1 refers to the pre-pandemic baseline reporting period of February/March 2020, while Time 2 and Time 3 refer to the mid-pandemic periods of early May and August 2020, respectively. Error bars represent the lower and upper limits of a 95% confidence interval; they are slightly dodged as to not overlap for improved clarity.

Estimated marginal means by gambling outcome appear in Figure 1. Results from the linear mixed model evaluating the main effect of timepoint on G-TLFB Duration (total minutes spent gambling), Frequency (total days spent gambling), and Expenditure (total amount of money spent gambling) per 14-day G-TLFB reporting period appear in Table 2. Consistent with hypotheses, there was a significant negative main effect of timepoint between Time 1 and Time 2 and between Time 1 and Time 3, and a positive main effect of timepoint between Time 2 and Time 3, across all outcomes (see Table 2). The one exception was a non-significant positive effect of timepoint (*p* = 0.071) between Time 2 and Time 3 for duration.

**TABLE 2:** Linear mixed-model results for overall gambling outcomes.

	Duration		Frequency		Expenditure	
	*b*	95% CI	*b*	95% CI	*b*	95% CI
Time 2 (May)^a^	−175.33***	−248.59; −102.08	−3.03***	−3.86; −2.21	−191.16**	−304.56; −77.77
Time 3 (August)^a^	−141.76***	−213.27; −70.26	−1.97***	−2.70; −1.24	−135.98**	−227.40; −44.56
Time 3 (August)^b^	33.57	−2.94; 70.08	1.07***	0.55; 1.58	55.18*	13.36; 97.01
**Demographics**						
Age	6.90*	0.57; 13.23	0.06	−0.02; 0.13	3.85	−1.56; 9.25
Gender	58.29	−4.86; 121.44	−0.29	−1.04; 0.46	17.24	−36.64; 71.11
Marital status	−36.74	−97.75; 24.28	−0.08	−0.81; 0.65	0.49	−51.56; 52.55
Recruitment source	−8.92	−87.53; 69.69	−0.80	−1.74; 0.13	15.15	−51.92; 82.21
**Gambling**						
PGSI	9.56**	3.53; 15.59	0.01	−0.06; 0.08	10.22***	5.07; 15.37
Monthly spending	1.48	−11.34; 14.29	0.01	−0.14; 0.16	0.56	−10.37; 11.49
**MINI**						
Openness	60.66	−10.73; 132.05	0.60	−0.25; 1.45	42.57	−18.33; 103.48
Conscientiousness	−60.98**	−105.99; −15.96	−0.41	−0.95; 0.12	−35.06	−73.47; 3.35
Extraversion	−20.92	−59.51; 17.67	−0.39	−0.85; 0.07	−11.12	−44.04; 21.81
Agreeableness	31.07	−25.28; 87.42	0.41	−0.26; 1.08	14.31	−33.76; 62.39
Neuroticism	−2.24	−54.94; 50.45	0.17	−0.46; 0.80	12.35	−32.61; 57.30
**GMQ**						
Enhancement	19.14	−37.14; 75.41	0.03	−0.64; 0.70	−14.83	−62.85; 33.18
Social	59.71*	5.48; 113.93	0.55	−0.10; 1.20	45.84	−0.42; 92.10
Coping	31.18	−79.83; 17.48	0.16	−0.42; 0.74	−20.83	−62.33; 20.68
N participants	85		85		85	
Observations	255		255		255	
Pseudo *R*^2^	0.21		0.26		0.24	

*p < 0.05, **p < 0.01, ***p < 0.001. Significant effects (p < 0.05) are in bold. 95% CI values indicate the lower and upper limits of a 95% confidence interval. ^a^Reference point is pre-pandemic baseline. ^b^Reference point is Time 2 (May).

These results indicate that participants engaged in gambling for less time in minutes, on fewer days, and spent less money during the pandemic in May and August 2020 than they did before the pandemic in late February and early March 2020. They also engaged in greater levels on two of the three gambling indices (Frequency and Expenditure) when major sports leagues had resumed play in August 2020 relative to when sports leagues worldwide had suspended play in May 2020, though values remained lower in August 2020 than pre-pandemic levels, suggesting only a partial return to baseline of gambling behavior. The model predicted approximately 21% of the variability in gambling duration (*McFadden’s Pseudo R^2^* = 0.21), 26% of the variability in gambling frequency (*McFadden’s Pseudo R^2^* = 0.26), and 24% of the variability in gambling expenditure (*McFadden’s Pseudo R^2^* = 0.24).

### Main effects of time period on sports gambling outcomes

Results from linear mixed models evaluating the main effect of timepoint on G-TLFB Duration, Frequency, and Expenditure of sports gambling per 14-day G-TLFB reporting period appear in Table 3. Consistent with hypotheses, there was a significant negative main effect of timepoint between Time 1 and Time 2 and between Time 1 and Time 3, and a positive main effect of timepoint between Time 2 and Time 3, across all outcomes (see Table 3). These results indicate that participants engaged in sports gambling for less time in minutes, on fewer days, and spent less money during the pandemic in May and August 2020 than they did before the pandemic in late February and early March 2020. They also engaged in greater levels of all three gambling indices in August 2020 when major sports leagues had resumed play relative to May 2020 when sports leagues worldwide had suspended play, though values were significantly lower in August 2020 compared to pre-pandemic levels, indicating only a partial return to baseline. No evidence for substitution effects was found in that no interactions of non-sport gambling and time were evident. The model predicted approximately 22% of the variability in gambling duration (*McFadden’s Pseudo R^2^* = 0.22), 26% of the variability in gambling frequency (*McFadden’s Pseudo R^2^* = 0.26), and 28% of the variability in gambling expenditure (*McFadden’s Pseudo R^2^* = 0.28).

**TABLE 3:** Linear mixed-model results for sports gambling outcomes.

	Duration		Frequency		Expenditure	
	*b*	95% CI	*b*	95% CI	*b*	95% CI
Time 2 (May)^a^	−143.28***	−215.97; −70.59	−2.34***	−3.20; −1.49	−138.99*	−246.80; −31.18
Time 3 (August)^a^	−103.86**	−174.89; −32.83	−1.26**	−2.01; −0.51	−94.88*	−177.12; −12.65
Time 3 (August)^b^	39.42*	7.18; 71.66	1.08***	0.56; 1.60	44.11**	12.16; 76.06
**Demographics**						
Age	6.77**	2.44; 11.09	0.06	−0.00; 0.12	3.13**	0.87; 5.40
Gender	39.32	−3.69; 82.33	0.18	−0.44; 0.80	14.06	−8.65; 36.77
Marital status	−17.53	−59.22; 24.16	−0.16	−0.75; 0.43	−0.15	−22.02; 21.72
Recruitment source	−18.78	−72.40; 34.84	−0.64	−1.40; 0.12	−1.05	−29.32; 27.21
**Gambling**						
PGSI	4.31*	0.10; 8.52	−0.01	−0.07; 0.05	3.97**	1.66; 6.28
Monthly spending	0.07	−8.65; 8.80	0.01	−0.12; 0.13	−0.15	−4.74; 4.45
**MINI**						
Openness	61.86*	13.30; 110.42	0.68	−0.01; 1.37	21.52	−4.19; 47.24
Conscientiousness	−29.27	−60.30; 1.77	−0.18	−0.62; 0.26	−12.35	−28.66; 3.96
Extraversion	−1.91	−28.71; 24.89	0.03	−0.36; 0.41	2.73	−11.17; 16.64
Agreeableness	9.01	−29.40; 47.42	0.26	−0.28; 0.81	6.14	−14.08; 26.35
Neuroticism	6.20	−29.66; 42.07	0.03	−0.48; 0.55	−2.47	−21.46; 16.51
**GMQ**						
Enhancement	17.04	−21.24; 55.32	0.03	−0.51; 0.57	−6.76	−27.01; 13.49
Social	26.32	−10.77; 63.42	0.20	−0.33; 0.72	21.97*	2.23; 41.70
Coping	−13.04	−46.25; 20.16	0.29	−0.19; 0.76	−6.51	−24.02; 11.00
Non-sports gambling	−0.20	−0.55; 0.16	0.26	−0.12; 0.65	−0.03	−0.36; 0.30
Non-sports gambling × time 2^a^	0.10	−0.30; 0.51	−0.07	−0.56; 0.42	−0.01	−0.39; 0.37
Non-sports gambling × time 3^a^	−0.10	−0.52; 0.31	−0.24	−0.69; 0.21	−0.05	−0.33; 0.23
Non-sports gambling × time 3^b^	−0.21	−0.56; 0.14	−0.17	−0.64; 0.30	−0.04	−0.22; 0.15
N participants	85		85		85	
Observations	255		255		255	
Pseudo *R*^2^	0.22		0.26		0.28	

*p < 0.05, **p < 0.01, ***p < 0.001. Significant effects (p < 0.05) are in bold. 95% CI = values indicate the lower and upper limits of a 95% confidence interval. ^a^Reference point is pre-pandemic baseline. ^b^Reference point is Time 2 (May).

### Main effects of time period on non-sports gambling outcomes

Results from linear mixed models evaluating the main effect of timepoint on G-TLFB Duration, Frequency, and Expenditure of all non-sports related gambling activities appear in Table 4. A negative main effect of timepoint between Time 1 and Time 2 was observed across all outcomes, and between Time 1 and Time 3 on Duration (see Table 4). These results indicate that participants engaged in non-sports gambling for less time in minutes, on fewer days, and spent less money during the pandemic in May 2020 than they did before the pandemic in late February and early March 2020. They also reported spending less time gambling on non-sports related activities in August 2020 compared to before the pandemic. In contrast to sports gambling analyses, no significant main effect of timepoint between Time 2 and Time 3 was observed across any of the outcomes. Although there was a significant negative interaction effect of sports gambling expenditure and timepoint between Time 1 and Time 3, we found weak evidence to support a potential substitution effect. As the amount of money spent on sports gambling decreased in August 2020 relative to February/March 2020, the amount of money spent on non-sports gambling was unchanging over that same interval (see Figure 1). The model predicted approximately 22% of the variability in gambling duration (*McFadden’s Pseudo R^2^* = 0.22), 24% of the variability in gambling frequency (*McFadden’s Pseudo R^2^* = 0.24), and 20% of the variability in gambling expenditure (*McFadden’s Pseudo R^2^* = 0.20).

**TABLE 4:** Linear mixed-model results for non-sports gambling outcomes.

	Duration		Frequency		Expenditure	
	*b*	95% CI	*b*	95% CI	*b*	95% CI
Time 2 (May)^a^	−47.15**	−76.77; −17.53	−0.37*	−0.73; −0.02	−39.83*	−72.65; −7.02
Time 3 (August)^a^	−47.57**	−80.72; −14.42	−0.29	−0.68; 0.10	−27.84	−61.38; 5.70
Time 3 (August)^b^	−0.42	−18.28; 17.43	0.08	−0.15; 0.31	11.99	−11.83; 35.82
**Demographics**						
Age	0.57	−2.89; 4.03	−0.01	−0.05; 0.03	−1.30	−5.45; 2.85
Gender	−1.78	−35.91; 32.35	−0.37	−0.76; 0.01	−17.80	−58.75; 23.16
Marital status	−4.45	−37.17; 28.27	0.10	−0.27; 0.47	14.04	−25.06; 53.13
Recruitment source	5.91	−36.17; 47.99	−0.10	−0.58; 0.38	13.67	−36.90; 64.23
**Gambling**						
PGSI	2.31	−0.98; 5.61	0.03	−0.00; 0.07	5.87**	1.87; 9.86
Monthly spending	1.34	−5.51; 8.19	−0.00	−0.08; 0.08	2.05	−6.16; 10.27
**MINI**						
Openness	−5.63	−44.43; 33.17	−0.11	−0.54; 0.33	26.95	−19.03; 72.93
Conscientiousness	−12.88	−37.11; 11.35	−0.13	−0.40; 0.15	−18.08	−47.11; 10.96
Extraversion	−21.27*	−41.94; −0.60	−0.30*	−0.53; −0.06	−6.68	−31.44; 18.08
Agreeableness	24.03	−6.13; 54.18	0.10	−0.24; 0.45	3.50	−32.69; 39.68
Neuroticism	0.36	−27.85; 28.57	0.21	−0.11; 0.53	13.74	−20.10; 47.58
**GMQ**						
Enhancement	−6.66	−36.85; 23.53	−0.04	−0.38; 0.30	−17.04	−53.24; 19.15
Social	11.95	−17.24; 41.13	0.26	−0.07; 0.59	35.59*	0.50; 70.68
Coping	−0.48	−26.52; 25.57	−0.17	−0.47; 0.12	−12.41	−43.67; 18.85
Sports gambling	−0.01	−0.08; 0.06	0.06	−0.01; 0.13	−0.02	−0.08; 0.04
Sports gambling × time 2^a^	0.02	−0.10; 0.13	−0.04	−0.15; 0.06	−0.16	−0.43; 0.11
Sports gambling × time 3^a^	−0.04	−0.15; 0.08	−0.05	−0.14; 0.05	−0.14*	−0.28; −0.01
Sports gambling × time 3^b^	−0.06	−0.18; 0.07	−0.00	−0.11; 0.10	0.02	−0.27; 0.30
N participants	85		85		85	
Observations	255		255		255	
Pseudo *R*^2^	0.22		0.24		0.20	

*p < 0.05, **p < 0.01. Significant effects (p < 0.05) are in bold. 95% CI = values indicate the lower and upper limits of a 95% confidence interval. ^a^Reference point is pre-pandemic baseline. ^b^Reference point is Time 2 (May).

## Discussion

The present study investigated the effect of the suspension and subsequent resumption of play in major sports leagues worldwide during the COVID-19 pandemic on Canadian sports bettors’ gambling behaviors. In accordance with the availability hypothesis, it was predicted that a general decrease in both gambling frequency and intensity would be observed across all variables of interest between the baseline (pre-pandemic) reporting period in February/March 2020 and the second reporting period in May 2020 when accessibility to sports gambling opportunities were at their minimum, followed by a subsequent rebound in gambling frequency and intensity corresponding with the increased availability of sports gambling opportunities as major sports leagues resumed live play in August 2020.

With respect to changes in gambling activity, the general pattern of results was largely consistent with hypotheses. Based on retrospective recall, participants reported a significant decrease in their frequency, duration, and expenditure on any type of gambling (sports or non-sports) from the February/March baseline period to early May. The observed drop in these three gambling behaviors during the early stages of the pandemic in Canada is consistent with findings on gambling behavior during the COVID-19 pandemic in other countries (21). Moreover, this uniform decrease across gambling behaviors between baseline and May mirrors the results of Lund (17) in providing support for the predictions of the availability hypothesis in the case of decreased gambling availability, while extending these findings by showing similar results in the context of an unplanned decrease in gambling availability related to the COVID-19 pandemic.

Significant increases in gambling behaviors between May and August were also observed, as public health measures limiting the availability of both sports and non-sports gambling were eased, with some variation according to gambling type. Unsurprisingly, participants reported large, significant increases in their frequency, duration, and expenditure related to sports gambling activities, corresponding to the return of most major live sporting events by August 2020 (38). Conversely, participants reported no significant increases in their non-sports related gambling behaviors over this same period. This pattern of findings is largely supportive of hypotheses, with the exception of finding no significant difference between May and August with respect to the frequency, expenditure, and duration of non-sports gambling. A plausible explanation for why a significant difference was observed in non-sports gambling from baseline to May, but not from May to August, has to do with the fact that most land-based casinos in Eastern Canada were closed from mid-March to early October (45). Given that most of the present study’s sample were from Eastern Canada, the retrospective report timepoints used likely captured the initial decrease in the availability of non-sports gambling opportunities (casino closures) but not any substantial increase in availability between May and August, as casinos remained closed. It is possible that a rebound effect consistent with the general pattern of results would have been observed had non-sports gambling frequency been measured at a time-point after which casinos in Eastern Canada had reopened. Alternatively, these results may indicate sport gamblers focus quite exclusively on resuming their sport gambling when the opportunities resume, rather than engaging in more non-sports related gambling.

Notwithstanding the absence of a rebound effect in gambling outcomes on non-sport activities in the data, the partial rebound observed for all other variables provides support for the availability hypothesis. Notably, this rebound effect was only partial in nature for all variables on which it was observed, such that gambling frequency, time spent gambling, and expenditure were statistically higher at baseline even after this partial rebound in August. There are several possible explanations for this. First, while the August timepoint for reporting was chosen to capture the effect of a return in the availability of sports betting as well as overall gambling, this choice of time-period was nonetheless somewhat arbitrary. The present study’s findings of only a partial rebound in gambling behaviors are consistent with similar findings in different populations using different time periods and reporting methods (33, 46). Thus, it is possible the partial rebound effect observed could be evidence of a sustainable shift toward a new, lower baseline of gambling behaviors. It may be that the initial drop in gambling behaviors experienced during the early stages of the pandemic led participants to modify their desire to gamble accordingly, making them less sensitive to the increased availability of gambling opportunities in August. This would represent a significant, unintended positive side-effect of the pandemic in terms of public health, as involvement in gambling behaviors is positively associated with the experience of gambling related harms (47–49). Indeed, there is evidence on voluntary self-exclusion (VSE) supporting this idea. VSE programs allow individuals concerned about their gambling to voluntarily sign themselves up to be banned from accessing gambling venues (50). These programs appear to be effective in reducing gambling behaviors and improving the psychological wellbeing of those involved (51). Moreover, these programs have lasting effects on gambling-related cognitive distortions, gambling behaviors, and problem gambling symptoms that can persist even after the VSE term is complete (50, 52, 53). In terms of the results of the present study, the decreased accessibility of gambling during the early stages of the pandemic may have acted as a natural self-exclusion program for sports gamblers that could very well have lasting effects on their gambling behaviors and desire to gamble going forward. Follow-up research in the aftermath of the pandemic is necessary to evaluate if this observed decrease from baseline in gambling involvement is sustained, and if so, for how long.

### Substitution to non-sports gambling

A plausible outcome of the pandemic would be that sports gamblers would increase their participation in non-sports gambling activities that were still widely available online as a means of “substituting” their gambling consumption. The interaction effect observed indicates that while sports gambling expenditure decreased from time 1 to time 3, non-sports gambling expenditure remained the same from time 1 to time 3. Thus, participants were spending a relatively larger proportion of their money on non-sports gambling at time 3 relative to time 1 (which could represent substitution), but they were nonetheless still spending more on sports gambling than non-sports gambling at time 3 (inconsistent with substitution). Moreover, the data did not take the form of a classic substitution effect as the interaction was not observed at baseline vs. time 2 (when sports gambling availability was most restricted), and since no increase in absolute expenditure on non-sports gambling was observed during the pandemic restrictions. Though we did not observe such a predicted interaction from time 2 relative to time 1, detection of small interaction effects in psychological research in mixed-effects models usually require sample sizes of *N* > 300 (54). Given the final sample size of *N* = 85, the present study was likely underpowered to detect small interaction effects. Nonetheless, any interpretation of our observed interaction effect as evidence of substitution must be made with caution, as this effect was observed at one timepoint comparison (time 3 relative to time 1) and for only one outcome (expenditure).

Aside from the interaction effect observed with respect to relative changes in sports vs. non-sports gambling expenditure at time 3, results regarding changes in the frequency of sports-related and non-sports gambling during the early stages of the pandemic (between baseline and time 2) do not provide any evidence that a substitution from sports gambling to non-sports gambling occurred. The present study’s failure to find strong, consistent evidence of substitution toward non-sports gambling is not entirely unexpected, as the evidence for substitution in the literature is relatively limited, with most studies finding little or no evidence suggestive of substitution to alternative forms of gambling following restricted availability of gambling opportunities (17, 24, 27). However, a study by Close et al. (55) found evidence of a large, significant increase in video-gaming involvement and problem gaming scores during the first lockdown period in the UK; this suggests some gamblers substituted their gambling consumption by increasing involvement in another potentially harmful and addictive activity. This notion is supported by a study conducted by Xuereb et al. (56), who found that while gambling involvement significantly decreased during the first lockdown period, drug use (alcohol, tobacco, cannabis) and other potentially addictive behaviors (video-gaming, pornography use) significantly increased over this same period.

### Implications for public health policy and treatment

The present study’s findings have implications for public health policy, providing support for findings connecting the availability of gambling opportunities to gambling outcomes, particularly with respect to sports gambling. The fact decreases in gambling involvement were observed across all gambling behavior outcome variables of interest speaks to the strength of the association between availability of sports gambling opportunities and degree of gambling involvement. Given that greater gambling involvement contributes to greater population level harms (49), the results from the present study suggest there is a case to be made for decreasing the availability of gambling in Canada as a means of reducing the significant public health burden of problem gambling (57). One way this could be achieved is through legislation designed to reduce accessibility of unregulated online gambling providers hosted in other countries that up until recently dominated the market for single event sports betting in Canada (58). Unfortunately, the current trajectory appears to be headed in the opposite direction, as the recent passing of Bill C-218 in the Canadian House of Commons paved the way for a legal single event sports betting market in Canada, thus greatly increasing the availability of sports gambling in the country. This may lead to greater gambling involvement and, in turn, higher rates of disordered gambling. It is important policymakers consider the potential negative consequences of expanded gambling availability and implement measures to balance out the expansion of legal sports gambling, such as by placing limits on offshore gambling providers.

In terms of clinical implications for treatment, results of the present study lend theoretical support to the efficacy of stimulus control methods to reduce the availability of gambling such as VSE programs in the treatment of problem gambling and gambling disorder. The present study’s finding of a substantial drop across all gambling behavior outcomes measured provides naturalistic evidence that the external imposition of strong restrictions to one’s access to gambling opportunities leads to large decreases in gambling behaviors associated with problem gambling severity (41). Given that substandard enforcement of VSE at gambling venues has been shown to lead to poorer treatment outcomes (51), the present study’s results suggest that stronger efforts to identify self-excluded gamblers and enforce their bans from access to gambling venues could lead to more potent treatment effects for VSE programs. Similarly, the present study’s findings support other stimulus control methods in the treatment of problem gambling, such as strictly enforced spending limits that are difficult to remove once gamblers have voluntarily agreed to enter a spending restriction program (53).

### Limitations

One of the primary limitations of the present study pertains to the length of time elapsed between retrospective report periods and actual data collection. Given that data were collected between September and early December of 2020, participants were required to report on their day-to-day gambling behaviors from between 1 and 3 months in the past for the August reporting period, between 4 and 6 months in the past for the May reporting period, and between 7 and 9 months in the past for the pre-pandemic baseline reporting period. The psychometric properties of the G-TLFB have only been evaluated for use in recall periods up to 6-months in the past (36). Though Weinstock et al. found the past 6-month recall version of the G-TLFB to exhibit strong reliability comparable to that of a past 3-month version of the G-TLFB, it is difficult to dispute the contention that accuracy of recall will diminish over more temporally distant periods. Interestingly, when evaluating the convergent validity of the G-TLFB with a daily diary measure of the same gambling behaviors, Weinstock et al. (36) found that the G-TLFB led to consistent underreporting of gambling outcomes. The fact that outcomes measured by the G-TLFB in the present study were highest at baseline, the most temporally distant reporting period, does allay some of the concerns about recall accuracy that arise from using this measure somewhat past its validated reporting length of 6 months. Nonetheless, results from the present study, particularly those from baseline data, must be interpreted with caution given the potential for retrospective recall bias.

Another limitation of the present study is our sample characteristics. As discussed, the relatively small sample size of this study indicates that we were likely significantly underpowered for detecting interaction effects in our model, which may have hindered our ability to detect substitution effects. Moreover, a small sample size restricted our ability to investigate possible substitutions between forms of sports related gambling that differed in their availability during the initial lockdown phase (e.g., from betting on team sports to e-sports to horse racing). Low sample size, coupled with our method of convenience sampling from a mixed population of adults in the community and university students, casts doubt on the generalizability of our results to the overall population of Canadian gamblers.

## Future directions and conclusion

There is much that remains unknown about how the ongoing COVID-19 pandemic has affected gambling behaviors. One area for future research is the identification of factors that moderate the relationship between gambling availability and gambling behaviors. Though existing research indicates most people gambled less in response to the pandemic, a subset of individuals responded by increasing their gambling, and these individuals may experience more psychological distress and engage in more risky behaviors (34, 59–61). It is important to identify how these individuals differ from the majority who reduce their gambling, to facilitate early identification and intervention for those whose mental health has been negatively affected during the pandemic. As noted by Claesdotter-Knutsson and Håkkanson (61), baseline problem gambling severity and psychological distress scores may be associated with an increase in gambling during the pandemic. However, a study by Gainsbury et al. (62) found only moderate risk gamblers increased their gambling during the pandemic and these authors reported no association between psychological distress and increased gambling, highlighting the need for additional research on this topic. Additional variables that are good candidates for investigation as potential moderators include coping motives, trait impulsivity, and neuroticism, as these have all been well established in the literature as risk factors for the development of problem gambling (63–65) and thus might similarly confer vulnerability to resistance to decreasing gambling even when gambling opportunities become less available.

Additionally, there is a need for continued longitudinal research to observe trajectories of change in gambling behaviors over the later stages and in the aftermath of the pandemic. Currently, Fluharty et al. (25), Månsson et al. (29), Biddle et al. (33), and the present study are the only known investigations of gambling behaviors that allow for comparisons between a pre-pandemic baseline and multiple periods corresponding to both early and later stages of the pandemic, and the present study is the first to extend this work to a population of Canadian gamblers.

Lastly, the evidence with respect to the existence or absence of a substitution from sports gambling to other forms of gambling amidst restricted availability remains inconclusive. Research is needed to determine whether sports gamblers substituted toward other forms of gambling in response to reduced availability of gambling opportunities during the pandemic. This research should also examine if gamblers are instead substituting gambling behaviors with substance use (e.g., alcohol, or cocaine) or other potentially addictive behaviors (e.g., excessive internet gaming, pornography use), in essence swapping one addictive activity for another. This concept of “addiction substitution” has been theorized (66), and is consistent with findings of increases in substance use and non-gambling related addictive behaviors during lockdown (55, 56).

The present study contributes to the literature concerning the impact of the COVID-19 pandemic on gambling by presenting the first known examination of dynamic changes in gambling behaviors across multiple stages of the pandemic in a Canadian sample. The present study replicated and extended the literature by demonstrating both an initial decrease and later partial rebound on a range of sports gamblers’ gambling behaviors during the pandemic, and in doing so provided support for the utility of the availability hypothesis in predicting change in gambling behavior as a function of gambling opportunities. Though the present study’s failure to find strong, consistent evidence of a shift from sports gambling to non-sports gambling over the course of the pandemic may allay concerns that the pandemic introduced sports gamblers to potentially riskier forms of gambling, continued monitoring of post-pandemic trends will be essential to fully understanding the lasting impact of the COVID-19 pandemic on gambling habits.

## Data availability statement

The raw data supporting the conclusions of this article will be made available by the authors, without undue reservation.

## Ethics statement

This study involving human participants was reviewed and approved by Health Sciences Research Ethics Board, Dalhousie University, REB#: 2020-5044. Written informed consent was received from all participants prior to their completion of study measures.

## Author contributions

EO: responsible for project conceptualization, survey construction, data collection, conducting analyses, literature review, writing entire first draft of manuscript, and introduction/discussion of final manuscript. AJK: responsible for conducting analyses and writing methods/results of final manuscript. SHS: EO’s research supervisor, collaborated with EO and IY for project conceptualization, heavily involved in decisions regarding data analyses, and editing of manuscript. SBS: AJK’s research supervisor, assisted AJK with analyses/provision of supervision related to project, and involved in editing manuscript. IY: EO’s research supervisor, collaborated with EO and SHS in project conceptualization, provision of research supervision to EO throughout project completion, including advising on writing of manuscript and consultation with respect to statistical methods necessary for data analysis, heavily involved in data collection, analyses, and editing of manuscript. All authors contributed to the article and approved the submitted version.
